# Unique expression features of cancer-type organic anion transporting polypeptide 1B3 mRNA expression in human colon and lung cancers

**DOI:** 10.1186/s40169-014-0037-y

**Published:** 2014-11-18

**Authors:** Yuchen Sun, Tomomi Furihata, Seiya Ishii, Miki Nagai, Manami Harada, Osamu Shimozato, Takehiko Kamijo, Shinichiro Motohashi, Ichiro Yoshino, Atsuko Kamiichi, Kaoru Kobayashi, Kan Chiba

**Affiliations:** 1 Laboratory of Pharmacology and Toxicology, Graduate School of Pharmaceutical Sciences, Chiba University, 1-8-1 Inohana, Chuou-ku, Chiba-shi 260-8675, Chiba, Japan; 2 Division of Biochemistry and Molecular Carcinogenesis, Chiba Cancer Center Research Institute, Chiba, Japan; 3 Department of Medical Immunology, Graduate School of Medicine, Chiba University, Chiba, Japan; 4 Department of General Thoracic Surgery, Graduate School of Medicine, Chiba University, Chiba, Japan

**Keywords:** OATP1B3, SLCO1B3, Colon cancer, Lung cancer, Cancer biomarker, Cancer-specific expression, Transporter

## Abstract

**Background:**

We have previously identified the cancer-type organic anion transporting polypeptide 1B3 (Ct-OATP1B3) mRNA in several human colon and lung cancer tissues. Ct-OATP1B3 is a variant of the liver-type OATP1B3 (Lt-OATP1B3) mRNA, which is a hepatocyte plasma membrane transporter with broad substrate specificity. However, in cancer tissues, both the detailed characteristics of Ct-OATP1B3 mRNA expression and its biological functions remain unclear. With this point in mind, we sought to characterize Ct-OATP1B3 mRNA expression in colon and lung cancer tissues. In addition, we attempted to obtain functional implication of Ct-OATP1B3 in cancer cells.

**Methods:**

Matched pairs of cancer and normal tissues were collected from 39 colon cancer and 28 lung cancer patients. The OATP1B3 mRNA expression levels in each of these tissues were separately determined by quantitative real-time polymerase chain reaction. Mann–Whitney U test and Fisher’s exact test were used in statistical analysis. The Ct-OATP1B3 functional expression in colon cancer cells was then examined by Western blotting and transport analyses.

**Results:**

Ct-OATP1B3 mRNA, but not Lt-OATP1B3 mRNA, was abundantly expressed in colon cancer tissues at a higher detection frequency (87.2%) than that of the adjacent normal tissues (2.6%). Furthermore, it was found that Ct-OATP1B3 mRNA expression was often detected in early colon cancer stages (88.9%, n = 18), and that its expression was associated with well-differentiated colon cancer statuses. On the other hand, Ct-OATP1B3 mRNA also showed a predominant and cancer-associated expression profile in lung tissues, although at frequencies and expression levels that were lower than those obtained from colon cancer. As for attempts to clarify the Ct-OATP1B3 functions, neither protein expression nor transport activity could be observed in any of the cell lines examined.

**Conclusions:**

Based on the unique characteristics of the Ct-OATP1B3 mRNA expression profile identified in this study, Ct-OATP1B3 mRNA can be expected to become a biomarker candidate for use in colon (and lung) cancer diagnosis. Simultaneously, our results advance the possibility that Ct-OATP1B3 might play yet unidentified roles, in addition to transporter function, in cancer cell biology.

## Background

It has been acknowledged that early detection and appropriate treatment are essential for overcoming the high mortality and morbidity of cancer, and recent advances have provided excellent results showing that utilization of cancer-associated molecules in cancer diagnosis and cancer therapy can contribute significantly to the development of more sensitive and accurate detection methods, as well as to the improvement of treatment outcomes in various types of cancer. As part of these extensive ongoing research efforts aimed at identifying molecules closely associated with cancer, the specific aberrant mRNA products found in cancer cells have attracted significant levels of attention [1]-[3]. Recent findings have shown that those aberrant gene products are among the hallmarks of cancer, and that they often play a role in an oncogenic pathway, as exemplified by the association of the human epidermal growth factor receptor 2 splicing variant with breast cancer cell invasion and trastuzumab resistance [4]. Thus, the characterizations of cancer-associated alternative mRNA products offer clear opportunities not only for development of diagnostic, prognostic, and therapeutic methods for cancer treatment, but also for gaining new insights into cancer biology.

Recently, we have made our first report on the identification of the cancer-type organic anion transporting polypeptide 1B3 (Ct-OATP1B3, [GenBank: NM_019844 (for SLCO1B3, gene symbol) and GenBank: AB669023 (for the alternative region)] in human colon and lung cancers [5], and other research groups have subsequently confirmed its existence in colon and pancreas cancers [6],[7]. Ct-OATP1B3 is an mRNA variant of the liver-specific OATP1B3 (which is hereafter referred to as the liver-type OATP1B3, Lt-OATP1B3) that mediates cellular uptake of a variety of endogenous compounds (e.g., cholecystokinin-octapeptide sulfated, CCK-8, or estradiol-17β-D-glucuronide, E_2_G), as well as drugs (e.g., paclitaxel or imatinib) at the basolateral membrane of hepatocytes [8],[9]. Previously, the detection of OATP1B3 (either or both Ct- and Lt-OATP1B3) has been reported in various cancer tissues, and it had been presumed that the mRNA was identical to Lt-OATP1B3 [10]-[15]. However, in contrast with this presumption, our investigations, along with others, have revealed that Ct-OATP1B3 mRNA expression is strongly associated with cancerous colon and lung tissues, but not with matched normal tissues or hepatocytes, and that Ct-OATP1B3 mRNA is expressed predominantly over Lt-OATP1B3 mRNA in these cancer tissues [5]-[7]. Therefore, those findings have shown that Ct-OATP1B3, but not Lt-OATP1B3, is a primary mRNA isoform, at least in these cancer tissues, which opens up the possibility of it being an intriguing cancer-associated molecule that can be used in the development of cancer biomarkers or therapeutic targets. Nevertheless, our current understanding of Ct-OATP1B3 mRNA expression profile in cancer tissues remains premature due to a small number of cancer tissues examined to date. Therefore, step-by-step characterization of Ct-OATP1B3 mRNA expression using a larger cohort will be required in order to validate and increase the feasibility of its clinical application.

**Figure 1:**
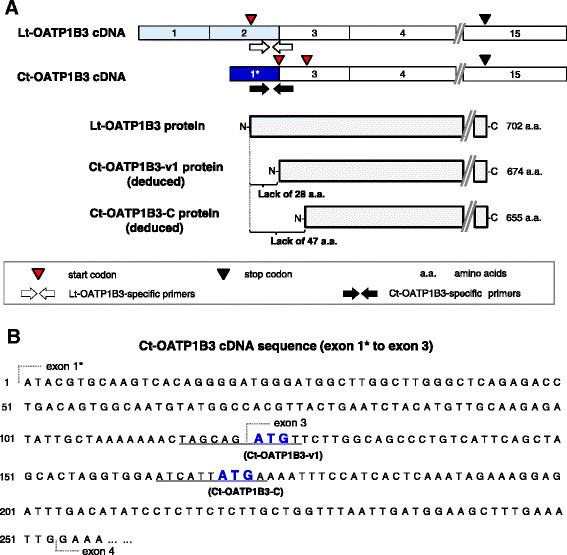
**Schematic illustration of the Ct-OATP1B3 cDNA structure and its predicted translation products. (A)** The cDNA structures of Ct- and Lt-OATP1B3 along with their predicted translation products were shown. The sky-blue boxes indicate the Lt-OATP1B3-specific exons, while the blue box indicates the Ct-OATP1B3-specific exon. The white boxes indicate the common exons. Each exon number is shown in the box. The translation product of Lt-OATP1B3 or the predicted translation products of Ct-OATP1B3 (Ct-OATP1B3-C and Ct-OATP1B3-v1) are shown below the cDNA structures. The red and black triangles indicate the translation start and stop codons, respectively. The open and closed arrows indicate an isoform-specific primer set for Lt-OATP1B3 or Ct-OATP1B3 detection, respectively. **(B)** The 5’-end cDNA sequence of Ct-OATP1B3 (exon 1* to 3) was shown. The bold blue letters indicate the predicted translation start codon (ATG) of Ct-OATP1B3-C or Ct-OATP1B3-v1. The underlines indicate the key sequences supposedly determining translation efficiency, while the consensus Kozak sequence is 5’-A/GNNatgG-3’, where small “atg” is the translation start codon.

Another important issue that needs to be addressed is clarification of the Ct-OATP1B3 function in cancer cells. Because the Ct-OATP1B3 mRNA transcription start site is located within the intron 2 of the *SLCO1B3* gene [5], Ct-OATP1B3 mRNA lacks the region that corresponds to the Lt-OATP1B3 N-terminal coding region. Therefore, the distinctive translation start codon usage is likely to occur in Ct-OATP1B3 mRNA (Figure 1). Our original research predicted a transporter-like translation product, which we termed Ct-OATP1B3-C [5]. Although, based on its deduced structure, Ct-OATP1B3-C is still expected to have a transporter function like Lt-OATP1B3, its functional expression remains to be demonstrated. Meanwhile, other studies have predicted the abilities of another product, Ct-OATP1B3-v1. However, Thakkar et al. [6] have reported that Ct-OATP1B3-v1 showed limited CCK-8 transport activity owing to its predominant localization in the cytoplasmic fraction, whereas Imai et al. [7] reported the cell surface expression of Ct-OATP1B3-v1 with transporting activities against fluvastatin and rifampicin. Therefore, additional examinations are absolutely essential to settling these controversies.

Based on all above-mentioned circumstances, this study aimed at characterizing the Ct-OATP1B3 mRNA expression profile using a larger number of colon and lung cancer tissue specimens, while simultaneously exploring its application potential as a cancer biomarker. Furthermore, we report on our attempt to identify a transporter-like entity derived from Ct-OATP1B3 mRNA.

## Methods

### Human-derived materials

Thirty-nine matched-pairs of human colon cancer and adjacent normal tissues were obtained from colon cancer patients who had undergone surgery (from June 2011 to June 2013) at the Chiba Cancer Center Research Institute (Chiba, Japan). Twenty-eight matched-pairs of human lung cancer and adjacent normal tissues were obtained from lung cancer patients who had undergone surgery (from January 2010 to February 2012) at the Chiba University Hospital (Chiba, Japan). The patient information is shown in Tables 1 and 2. The source of human liver tissue was described in our previous research [16]. Fifty donor-pooled human hepatocytes (Hep50) were purchased from Celsis IVT (Baltimore, MD). Use of human samples in this study was approved by the Ethics Committees of Graduate Schools of Pharmaceutical Sciences and Medicine, Chiba University, and the Ethics Committee of the Chiba Cancer Center Research Institute. Written informed consent was obtained from each patient.

**Table 1:** Demographic and clinical characteristics of colon cancer patients

**Variable**		**N (total = 39)**
Age at surgery (year)	Median	67
	Range	41-84
Sex	Male	30
	Female	9
Location	Cecum	3
	Ascending colon	5
	Transverse colon	1
	Descending colon	2
	Sigmoid colon	11
	Rectum	17
Stage	0	1
	I	2
	II	15
	III	12
	IV	4
	Unknown	5
Differentiation	Well	10
	Moderate	21
	Unknown or unclassified	8

**Table 2:** Demographic and clinical characteristics of lung cancer patients

**Variable**		**N (total = 28)**
Age at surgery	Median	67
	Range	44-81
Sex	Male	20
	Female	8
Cancer type	Adenocarcinoma	12
	Squamous cell carcinoma	12
	Adenosquamous carcinoma	1
	Small cell carcinoma	1
	Large cell carcinoma	2
Stage	I	20
	II	6
	III	2
Brinkman index^a^	<400	5
	>400	23

^a^Brinkman index was calculated as the product of the number of cigarettes smoked per day multiplied by the number of years of smoking.

The tumor stages of the colon and lung cancer patients were determined using the Union for International Cancer Control (UICC) tumor-node-metastasis (TNM) classification system. The differentiation status of colon cancer tissues was determined by pathological examination.

### Cells and cell culture

LS180 and HCT116 (colon cancer) cells were obtained from DS Pharma Biomedical (Osaka, Japan) and Dr. B. Vogelstein (Johns Hopkins University, Baltimore, MD), respectively.

LS180 cells were cultured in Eagle's Minimum Essential Medium (Wako, Osaka, Japan). HCT116 cells were maintained in Dulbecco's Modified Eagle's Medium (Wako). All culture mediums were supplemented with 10% (v/v) heat-inactivated fetal bovine serum and antibiotics. The cells were grown at 37°C with 5% CO_2_.

### Total RNA isolation and cDNA synthesis

Total RNA isolation from the human tissues and cells was performed using an ISOGEN II (NipponGene, Tokyo, Japan) according to the manufacturer’s protocol. cDNA synthesis from extracted RNA (1 μg) was performed using random hexamers as described previously [5].

### Quantitative real-time polymerase chain reaction (qPCR)

qPCR was performed using the methods described in our previous report [5]. Briefly, a SYBR green-based method was used for Lt-OATP1B3 mRNA expression quantification with the primer set (5’-AACAGCAGAGTCAGCATCTTCAG-3’ and 5’-AACATCTTGAATCCATTGCAGC-3’). Ct-OATP1B3 mRNA expression level was determined using a fluorescent probe-based method with the primer set (5’-TTGGCTTGGGCTCAGAGA-3’ and 5’-TGCCAAGAACATCTGCTAGTTT-3’), and universal probe #59 (Roche, Basel, Switzerland). The cDNA standard curve for each OATP1B3 mRNA was synthesized from each OATP1B3 mRNA at different copy numbers (from 10^3^ to 10^8^ copies for Ct-OATP1B3, and from 10^3^ to 10^6^ copies for Lt-OATP1B3). Each OATP1B3 expression level (copies/ng total RNA) in colon tissues, lung tissues, and cancer cell lines was calculated using its corresponding standard curve based on the condition that the mRNA expression level below 10^3^ copies/ng total RNA, which was regarded as the level below the quantification limit (QL), the qPCR Ct-value of which was over 35. The mRNA expression levels over QL were regarded as positive expression (or positive patients), whereas those under the QL were regarded as negative expression (or negative patients).

The total OATP1B3 mRNA (Ct- plus Lt-OATP1B3) and glyceraldehyde 3-phosphate dehydrogenase (GAPDH) mRNA levels were respectively determined using universal probe #59 with the primer set (5’-CGGCCTAACCTTGACCTATG-‘3 and 5’-TGAGTTGCAATAAGAAAGTGGTACA-3’), and probe #60 with the primer set (5’-AGCCACATCGCTCAGACAC-‘3 and 5’-GCCCAATACGACCAAATCC-3’). Data were calculated using the delta-delta-Ct method, where the GAPDH mRNA levels were used as a normalization control.

### Western blot analysis

Whole cell lysates were prepared from each cancer cell line using the lysis buffer or the Tris/Sucrose/EDTA buffer. The soluble membrane fractions were prepared from the whole cell lysates. Briefly, after centrifugation at 1,000 × *g* for 10 min, the supernatant was ultracentrifuged at 100,000 × *g* for 45 min. Then, the soluble membrane fractions were collected by dissolving the precipitates with the Tris/Sucrose/EDTA buffer supplemented with 0.8% (v/v) Nonidet P-40, 0.4% deoxycholic acid, and 0.08% sodium dodecyl sulfate (SDS), followed by a second ultracentrifugation under the same conditions. Using the same procedure, the soluble membrane fraction of human liver tissue was prepared.

The proteins were separated by SDS-polyacrylamide gel electrophoresis, and then transferred onto a polyvinylidene difluoride membrane. The membrane was blocked with 5% skim milk for one hour at room temperature. Affinity purified rabbit polyclonal anti-OATP1B3 antibodies, which recognize the C-terminal region of Lt-OATP1B3, were used as the primary antibodies (1,000-fold dilution, HPA004943, Sigma, St. Louis, MO). Anti-Na^+^/K^+^ ATPase (1,000-fold dilution, Sigma) and anti-β-actin (500-fold dilution, Sigma) were also used. Immunocomplexes were detected with ECL Western blotting detection reagents (GE Healthcare, Giles, UK).

### Transport assays

[^3^H]-CCK-8 and [^3^H]-E_2_G were obtained from PerkinElmer (Boston, MA), while non-radiolabeled CCK-8 and E_2_G were purchased from the Peptide Institute (Osaka, Japan) and Sigma, respectively. The transport assay was performed using LS180 and HCT116 cells based essentially on the previously described method [17]. Final concentrations of CCK-8 and E_2_G were 1 μM and 0.5 μM, respectively. The transport activity for each substrate was determined within a linear range (5 min). In the inhibition analysis, bromosulfophthalein (BSP, Sigma) (100 μM) was used.

### Statistical analysis

Mann–Whitney U test was used to determine differences of mRNA expression levels between two groups. Fisher’s exact test was used to determine the univariate relationship of the frequency of Ct-OATP1B3 or Lt-OATP1B3 mRNA expression between normal and cancer tissues. In all of the comparative analyses, only the positively detected expression data were used. All statistical analyses were performed using the Statcel software (OMS Publishing Inc., Tokyo, Japan).

### Others

Detailed development methods for the Ct-OATP1B3-C and Ct-OATP1B3-v1 expression plasmids, development of human embryonic kidney (HEK) 293 cells transiently or stably expressing each OATP1B3 isoform, as well as anti-OATP1B3 rabbit serum preparation, are described in Additional file 8 (supplemental materials and methods). Experimental procedures for the Lt-OATP1B3 expression plasmid and immunocytochemistry were described in the previous report [18].

## Results

### Expression profile of each OATP1B3 mRNA isoform in human colon cancer

**Figure 2:**
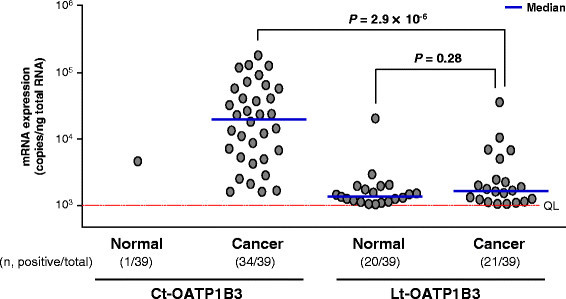
**Expression profiles of Ct- and Lt-OATP1B3 mRNA in human colon cancer patients.** The copy numbers of two OATP1B3 mRNA isoforms in each sample were separately determined by qPCR using the isoform-specific primers. Each dot represents the mean of Ct- or Lt-OATP1B3 mRNA expression levels (copies/ng total RNA), which was obtained from three independent determinations, each performed in duplicate. The red line indicates the value of the quantification limit (QL). The total number of specimens, together with the number of positive expression specimens, in each group is shown in parentheses. Horizontal solid lines denote the median value of positively detected mRNA levels in each group. The statistical significance of median difference between two groups was examined using the Mann–Whitney U test.

Ct-OATP1B3 and Lt-OATP1B3 mRNA quantification was performed using isoform-specific primer sets. The results showed that Ct-OATP1B3 mRNA expression frequencies in cancer and normal tissue specimens were 87.2% (34/39) and 2.6% (1/39), respectively (Fisher’s exact test, *P* = 1.24 × 10^−15^) (Figure 2). In addition to this high positive frequency, the mRNA levels in cancer tissues were strikingly higher than those in normal tissues (Figure 2). Accordingly, the apparent tumor/normal expression ratio (the T/N ratio) of Ct-OATP1B3 mRNA in each tissue pair was very high (Additional file 1: Figure S1A), even though the exact T/N ratios could not be calculated due to the quantitatively undetectable Ct-OATP1B3 mRNA levels in most normal tissues.

In contrast to the cancer-specific expression of Ct-OTP1B3 mRNA, Lt-OATP1B3 mRNA expression frequencies in cancer and normal tissue specimens were 53.8% (21/39) and 51.3% (20/39), respectively (Fisher’s exact test, *P* = 0.5) (Figure 2). The median values of Lt-OATP1B3 mRNA levels were similar between cancer and normal tissues (Figure 2), and were strikingly lower than those in human pooled hepatocytes (Table 3).

**Table 3:** Ct-OATP1B3 and Lt-OATP1B3 mRNA levels in colon tissues and pooled human hepatocytes

**mRNA name**	**Tissue (n)**^**a**^	**Median value (25-75**^**th**^**percentile)(×10**^**3**^**copies/ng total RNA)**
Ct-OATP1B3	Cancer (34)	19.5 (5.3-50.2)
	Normal (1)	N/A^b^
	Pooled hepatocytes	N/A^c^
Lt-OATP1B3	Cancer (21)	1.6 (1.2-2.4)
	Normal (20)	1.4 (1.2-1.7)
	Pooled hepatocytes	56.9 ± 2.5^d^

^a^The number of Ct-OATP1B3 or Lt-OATP1B3 mRNA positive patients is shown in parentheses.

^b^The Ct-OATP1B3 mRNA copy number in the normal tissue is 4.3 × 10^3^ copies/ng total RNA.

^c^The Ct-OATP1B3 mRNA expression level in pooled hepatocytes is under the quantification limit.

^d^The Lt-OATP1B3 mRNA expression level in pooled hepatocytes is expressed as the mean value ± SD.

When the Ct- and Lt-OATP1B3 mRNA levels in cancer tissues were compared, the median value of Ct-OATP1B3 mRNA levels in cancer tissues was 12.2-fold higher than that of Lt-OATP1B3 mRNA levels (19.5 × 10^3^ vs. 1.6 × 10^3^ copies/ng total RNA, *P* = 2.9 × 10^−6^) (Figure 2). In addition, Ct-OATP1B3 mRNA levels were also higher than Lt-OATP1B3 mRNA levels in individual cancer specimens (Additional file 2: Figure S2A).

It should be noted that the similar Ct- and Lt-OATP1B3 mRNA expression results were obtained using a ΔΔCt-method with the GAPDH mRNA level used as a normalization control (data not shown).

Taken together, our data showed that Ct-OATP1B3 mRNA was expressed in a cancer tissue-specific manner, and that its mRNA expression was predominant over Lt-OATP1B3 mRNA expression in colon cancer tissues.

### Associations of Ct-OATP1B3 mRNA levels in colon cancer tissues with clinico-pathological variables

Exploration of the clinico-pathological characteristics of Ct-OATP1B3 mRNA expression profiles in colon cancer began with an examination of the association of Ct-OATP1B3 mRNA levels and expression frequencies with cancer stages. As the results show, the Ct-OATP1B3 mRNA expression frequencies in early (0, I, and II) and advanced (III and IV) cancer stages were 88.9% (16/18) and 93.8% (15/16), respectively (Table 4). The median Ct-OATP1B3 mRNA level tended to be higher in the early stages than that in advanced stages (22.9 × 10^3^ vs. 11.6 × 10^3^ copies/ng total RNA, Table 4), although this difference was not statistically significant (Mann–Whitney U test, *P* = 0.3).

**Table 4:** Ct-OATP1B3 mRNA levels in early and advanced stages of colon cancer tissues

**Cancer stage**	**Gene expression frequency**	**Gene expression levels**^**a**^
	**% (n, positive/total)**	**Median value (25-75**^**th**^**percentile)**
Early (0, I, and II)	88.9 (1/1, 2/2, and 13/15)	22.9 (11.1-62.2)
Advanced (III and IV)	93.8 (12/12 and 3/4)	11.6 (4.8-32.7)

^a^The value is expressed as × 10^3^ copies/ng total RNA.

**Figure 3:**
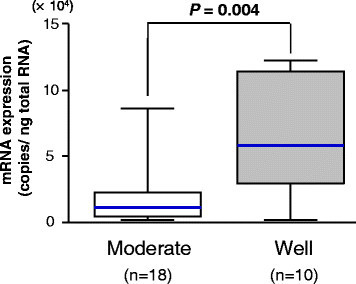
**Comparison of the Ct-OATP1B3 mRNA expression levels between well and moderately differentiated colon cancer tissues.** The colon cancer specimens with positive Ct-OATP1B3 mRNA expression were classified into two groups by the degree of differentiation, well-differentiated (n = 10), and moderately differentiated (n = 18). Data are shown as the box-and-whisker plot. The statistical significance of the median difference between two groups was determined using the Mann–Whitney U test.

Next, to test the association of Ct-OATP1B3 mRNA expression with the differentiation status of colon cancer, the Ct-OATP1B3 mRNA levels between well-differentiated and moderately differentiated cancer tissues were compared (poorly differentiated and undifferentiated cancer tissues could not be obtained for use in this study). The median value of Ct-OATP1B3 mRNA levels in well-differentiated cancer tissues was 5.2-fold higher than that in moderately differentiated cancer tissues (58.1 × 10^3^ vs. 11.2 × 10^3^ copies/ng total RNA, Mann–Whitney U test, *P* = 0.004) (Figure 3). It should be also noted that three out of five cancer tissue specimens that did not express Ct-OATP1B3 mRNA were moderately differentiated cancer (the degree of differentiation in the rest two specimens was unavailable) (data not shown).

Although the association of the Ct-OATP1B3 mRNA levels with other factors (age, sex and tumor location) was also examined, no statistically significant correlation could be found (data not shown).

### Expression profile of each OATP1B3 mRNA isoform in human lung cancer

**Figure 4:**
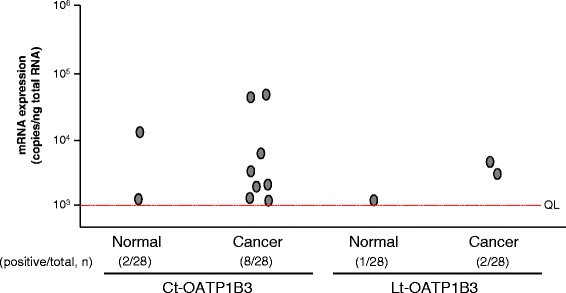
**Expression profiles of Ct- and Lt-OATP1B3 mRNA in human lung cancer patients.** Using the same quantification and calculation methods as described in the legend of Figure 2, the copy numbers of two OATP1B3 mRNA isoforms in each sample were separately determined. Each dot represents the mean of Ct- or Lt-OATP1B3 mRNA expression levels (copies/ng total RNA), which was obtained from three independent determinations, each performed in duplicate. The red line indicates the QL value. The total number of specimens, together with the number of positive expression specimens, in each group is shown in parentheses.

Ct- and Lt-OATP1B3 mRNA in human lung cancer was also separately quantified by qPCR. Compared with the results obtained from colon cancer tissues, the Ct-OATP1B3 mRNA expression frequency in lung cancer was relatively low (28.6%, 8/28) (Figure 4). Nevertheless, this frequency was statistically high compared with that obtained from normal lung tissues (7.1%, 2/28) (Fisher’s exact test, *P* = 0.039) (Figure 4), and the mRNA level in each cancer tissue was higher than that in the matched normal tissue (Additional file 1: Figure S1B).

Regarding Lt-OATP1B3 mRNA expression, its positive frequencies in cancer and normal lung tissues were 7.1% (2/28) and 3.6% (1/28) (Figure 4), and the Lt-OATP1B3 mRNA level was lower than the Ct-OATP1B3 mRNA level in each matched pair (Additional file 2: Figure S2B).

Again, the similar Ct- and Lt-OATP1B3 mRNA expression results were obtained using a ΔΔCt-method with the GAPDH mRNA level as a normalization control (data not shown).

### Comparison of Ct-OATP1B3 mRNA levels between colon and lung cancer

**Figure 5:**
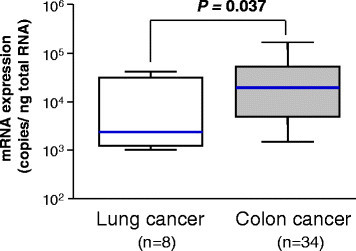
**Comparison of the Ct-OATP1B3 mRNA levels between lung and colon cancer tissues.** The Ct-OATP1B3 mRNA expression levels in lung (n = 8) and colon (n = 34) cancer tissues are shown as the box-and-whisker plot. Horizontal solid lines denote the median mRNA levels in each group. The statistically significant difference of the median Ct-OATP1B3 mRNA levels between two groups was examined using the Mann–Whitney U test.

When Ct-OATP1B3 mRNA levels between colon and lung cancer tissues were compared, it was found that the median value of Ct-OATP1B3 mRNA levels in colon cancer tissues was 8.1-fold higher than that in lung cancer tissues (19.5 × 10^3^ vs. 2.4 × 10^3^ copies/ng total RNA, Mann–Whitney U test*, P* = 0.037) (Figure 5).

### Functional expression analysis of endogenous Ct-OATP1B3 in colon cancer cell lines

**Figure 6:**
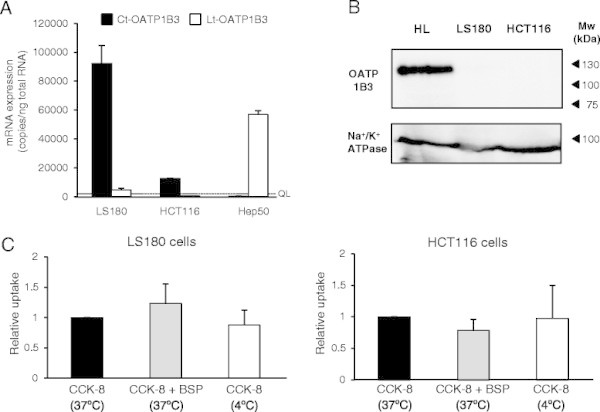
**Protein expression and functional analysis of Ct-OATP1B3 in colon cancer cell lines. (A)** Quantification of Ct- and Lt-OATP1B3 mRNA expression levels in human pooled hepatocytes (Hep50) and colon cancer cells (LS180 and HCT116) was separately performed by qPCR. The black and white bars indicate Ct- and Lt-OATP1B3 mRNA levels, respectively. The values are shown as means ± S.D. of three independent experiments, each performed in duplicate. The dashed line indicates the QL value. **(B)** The membrane protein expression of Ct-OATP1B3 in colon cancer cells was examined by Western blotting using rabbit anti-OATP1B3 antibodies that recognize the C-terminal peptide, which was supposed to be the common region among both isoforms. The soluble membrane fraction of human liver tissue (HL) was used as a positive control. Na^+^/K^+^ ATPase was used for a plasma membrane marker. **(C)** The CCK-8 uptake was examined by transport analysis using LS180 and HCT116 cells. The CCK-8 uptake (1 μM) level was determined in the absence (black bar) or presence (gray bar) of BSP (100 μM). The CCK-8 uptake in cancer cells is expressed as the relative ratio against the uptake of CCK-8 without BSP at 37°C. The white bar indicates transporter-independent CCK-8 accumulation level, which was determined at 4°C. This experiment was performed three times in duplicate.

The high Ct-OATP1B3 mRNA expression in cancer tissues highlights the possibility that it plays certain roles in cancer biology. Thus, the functional expression analysis of Ct-OATP1B3 was performed using human LS180 and HCT116 cells (colon cancer). The results of mRNA quantification showed that, similar to those observed in colon cancer tissues, Ct-OATP1B3 mRNA was highly expressed in LS180 and HCT116 cells, and that the mRNA level in LS180 cells was comparable with that of Lt-OATP1B3 in pooled hepatocytes (Table 3, Figure 6A).

Subsequently, endogenous Ct-OATP1B3 protein expression and functional analysis were performed by Western blotting and transport assays. Despite high Ct-OATP1B3 mRNA expression levels in colon cancer cells, its protein expression was not detected in the soluble membrane fraction of such cells when using either anti-OATP1B3 antibodies (which was obtained from Sigma) or anti-OATP1B3 rabbit serum (which was developed in this study) (Figure 6 and Additional file 3: Figure S3). Na^+^/K^+^ ATPase, which is a plasma membrane marker protein, was detected in all samples. Considering the possibility that Ct-OATP1B3 could be localized somewhere within intracellular fractions, the same analysis was also performed using whole cell lysates. However, no Ct-OATP1B3 protein expression was detected in any of those cells (Additional file 4: Figure S4). Consistently, CCK-8 transport activity was not detected in colon cancer cells, nor was this activity inhibited by BSP, which is a known OATP1B3 inhibitor (Figure 6C). Similar results were obtained from transport assay using E_2_G as a substrate (data not shown).

### Ct-OATP1B3 functional expression analysis in cells transfected with each Ct-OATP1B3 isoform expression plasmid

We further examined the functional expression of Ct-OATP1B3 using HCT116 cells transiently expressing each OATP1B3 isoform. The immunocytochemistry and transport assay results did not show any Ct-OATP1B3 protein expression or CCK-8 uptake activity in HCT116 cells transiently transfected with each Ct-OATP1B3 isoform (Additional file 5: Figure S5). However, Lt-OATP1B3 protein expression, as well as its transport activity, was clearly detected in the same experimental condition.

Similar results were also obtained from HEK293 cells stably expressing an OATP1B3 isoform (Additional file 6: Figure S6). As expected, Lt-OATP1B3 expression and function were clearly detected in HEK293 cells stably expressing Lt-OATP1B3. However, despite comparable mRNA levels among the cells, the results of Western blotting and transport assays failed to show functional Ct-OATP1B3 expression in any HEK293 cells expressing either Ct-OATP1B3-C or CT-OATP1B3-v1.

## Discussion

In agreement with our previous study, the results of the present study have shown that, even when using a larger number of the matched-pair tissue specimens, the Ct-OATP1B3 mRNA expression level in individual cancer tissue is always higher than the level in matched normal tissues, and is always predominant over the Lt-OATP1B3 mRNA level in each cancer tissue. These findings can be regarded as supporting evidence that Ct-OATP1B3 is the *bona fide* OATP1B3 mRNA isoform expressed in human colon and lung cancer. Thus, special attention should be paid to Ct-OATP1B3 expression (rather than Lt-OATP1B3) in future studies on cancer-associated OATP1B3. Furthermore, due to the high mRNA sequence similarities between the two isoforms, an elaborative experimental design is strongly recommended for such studies.

Cancer-specific RNA, DNA modification, or secreted molecules have been used as cancer biomarkers, such as carcinoembryonic antigen (CEA) and carbohydrate antigen 19–9 (CA19-9) in colon cancer [19]-[21]. In addition, numerous studies have been conducted to identify new clinically useful cancer biomarkers, as exemplified by Kallikrein-related peptidase 10 (KLK10) mRNA, which shows a cancer-specific profile [22]. Accordingly, the high positive rate (87.2%) of Ct-OATP1B3 mRNA expression in colon cancer tissues, along with its high T/N ratio, motivates us to evaluate it as a possible colon cancer biomarker candidate. Cancer biomarkers should possess sufficient and appropriate sensitivity and specificity that allows them to fulfill their roles in a given cancer therapy (cancer detection, metastasis or recurrence risk assessment, or response prediction). Therefore, in order to evaluate those properties, the area under the receiver operating characteristic curve analysis has been widely used (higher score is preferable with the maximum value = 1) [23]. A preliminary examination shows that the value of Ct-OATP1B3 mRNA is 0.93 for colon cancer diagnosis (Additional file 7: Figure S7). This value is comparable to that of serum CEA (0.86) or KLK10 mRNA (0.89) and higher than that of CA19-9 (0.58) [19],[22], suggesting that Ct-OATP1B3 mRNA may possess the clinically useful level of diagnostic power necessary to discriminate between cancer and normal colon tissues. In addition, the newly-identified clinico-pathological Ct-OATP1B3 mRNA expression features, which are its high rate of detection in early stages of colon cancer as well as its association with the well-differentiated cancer status, are considered noteworthy.

Based on the above considerations, it is reasonable to expect that Ct-OATP1B3 mRNA may be a highly promising candidate of colon cancer biomarker. However, we should reiterate the limitations of this study, which underscore the necessity of further investigations using an expanded cohort, various populations, and multicenter analyses in order to produce a comprehensive evaluation of its usefulness and restrictions. Elucidation of associations between Ct-OATP1B3 mRNA expression and prognostic values is another key issue that needs to be addressed (we were unable to pursue in this study due to use of recently obtained tissue specimens). Furthermore, it will be important to explore whether Ct-OATP1B3 mRNA can be detected in blood, because it has become evident that serum exosomes secreted from cancer cells contain cancer cell–derived molecules [24], such as the tumor-specific mRNA splicing variant detected in serum exosomes obtained from several glioblastoma patients [25].

Taken together, although extensive research efforts will be necessary before Ct-OATP1B3 mRNA can be established as a colon cancer biomarker, it is considered likely that such research will be worthwhile to promote in view of the urgent need for diagnostic tools in colon cancer, including the need for the development of a more reliable and less-invasive detection marker for patients in early stages of the disease. It has been acknowledged that examination of serum CEA and CA19-9 does not have sufficient capability for early identification of colon cancer due to their low abnormality rates in stage I (12-19% and 8%, respectively) and stage II patients (47-49% and 17%, respectively) [20],[21]. Therefore, once established, it is speculated that Ct-OATP1B3 mRNA, together with other markers and image diagnostic methods such as colonoscopy, computerized tomography, and magnetic resonance imaging, may improve the accuracy and sensitivity of current colon cancer screening, and may provide other important clinico-pathological information that ultimately contribute to reduction in the incidence of morbidity and mortality of the disease.

In addition to colon cancer, Ct-OATP1B3 mRNAs were also detected in about one third of lung cancer patients as well. This finding suggests that Ct-OATP1B3 mRNA may also be an indicator of lung cancer occurrence, although to a lesser degree. In line with the observations that Ct-OATP1B3 mRNA has been detected in other cancer types [6],[7], it will be necessary to clarify Ct-OATP1B3 mRNA expression preference in various cancer types to expand its clinical potential.

Functions of genes that are overexpressed in a cancer-specific manner are often involved in oncogenic processes [26]-[28]. Therefore, it is rational to assume that association of Ct-OATP1B3 mRNA expression with well-differentiation cancer status, as well as differential expression levels between lung and colon cancer, have some links with certain cancer cell biological processes.

Based on the predicted transporter-like structure of Ct-OATP1B3 translation products, which are Ct-OATP1B3-C and Ct-OATP1B3-v1 (Figure 1), one of the plausible functions of Ct-OATP1B3 is hormone uptake into cancer cells, as has been reported with the OATP1A2 function in prostate cancer [29]. The previous results provided by a cell-based exogenous Ct-OATP1B3-v1 transient expression system might also support this possibility [6],[7]. However, our results did not identify any functional expression of endogenous Ct-OATP1B3 in colon cancer cells, nor were we able to identify exogenous Ct-OATP1B3 function in HEK293 cells stably expressing Ct-OATP1B3-C or Ct-OATP1B3-v1. Thus, these results show apparent inconsistencies regarding the functional expression of Ct-OATP1B3. Because the experimental transport assay procedures employed in those studies are quite similar, the reason for the above-mentioned controversy is currently unclear. Therefore, further research aimed at providing convincing experimental evidence showing whether or not the Ct-OATP1B3 protein exists will be necessary to solve the above-mentioned arguments. However, in such future studies, we suggest taking into consideration the possibility that the translation efficiency of Ct-OATP1B3-v1 or Ct-OATP1B3-C might be very low. This is because the genomic DNA sequence around the Ct-OATP1B3-v1 start codon shows much less homology to the Kozak sequence (Figure 1B) [30], and because Ct-OATP1B3-C has a long 5’-untranslated region.

Nevertheless, we believe it is important to publicize our unexpected results pointing out that the transport function may not be the major role of endogenous Ct-OATP1B3 in cancer cells. This is because the results provide, rather than exclude, additional possibilities relating to how Ct-OATP1B3 plays a functional role in cancer cell biology. Recently, it has been shown that long non-coding RNA can directly interact with proteins to promote cancer metastasis [31], while another report has shown that a short cell-penetrating peptide derived from the Wilm’s tumor protein 1 has inhibitory effects on cancer proliferation and clonogenic activity [32]. In our previous report, the existence of Ct-OATP1B3-derived short peptides was suggested [5]. Therefore, while it is already clear that Ct-OATP1B3 plays far-reaching roles in cancer cells, numerous *in vivo* as well as *in vitro* experiments still remain to be conducted. The results of these experiments can be expected to provide important clues that will help identifying the functions of Ct-OATP1B3 protein, peptides, or the mRNA itself in cancer cell biology.

## Conclusion

Our results not only provide further evidence of the primary Ct-OATP1B3 mRNA expression profile in human colon and lung cancer, but also identify new clinico-pathological features of the Ct-OATP1B3 mRNA expression. Even though these results should be interpreted with caution due to several study limitations, it can nevertheless be said that Ct-OATP1B3 mRNA has the potential to become a promising biomarker candidate for colon (and lung) cancer diagnosis. On the other hand, our data suggests that, although existence of a transporter-like Ct-OATP1B3 protein cannot be fully excluded, it may not functionally active at detectable levels. This highlights the need to give serious consideration to any molecules potentially originating from the Ct-OATP1B3 gene in further investigations aimed at obtaining a more precise understanding of the roles played by Ct-OATP1B3 in cancer cells. We believe that such functional studies on Ct-OATP1B3 will provide new insights into cancer biology, while simultaneously enhancing translational research into its role as a cancer biomarker candidate.

## Abbreviations

BSP: Bromosulfophthalein

CCK-8: Cholecystokinin-octapeptide sulfated

Ct- and Lt-OATP1B3: Cancer-type and liver-type organic anion transporting polypeptide 1B3, respectively

E_2_G: Estradiol-17β-D-glucuronide

GAPDH: Glyceraldehyde 3-phosphate dehydrogenase

Hep50: Fifty donor-pooled human hepatocytes

QL: The quantification limit

qPCR: Quantitative real-time polymerase chain reaction

SLCO: Solute carrier organic anion transporter

T/N ratio: Tumor/normal expression ratio

## Competing interests

The authors declare that they have no competing interests.

## Authors’ contributions

YS, TF, and KC participated in the design of the study. YS, SI, MN, MH, and AK performed the experiments. OS, TK, SM and IY provided the research materials and analyzed the results. YS, TF, KK and KC analyzed the results and wrote the manuscript. All authors read and approved the final manuscript.

## Additional files

## Supplementary Material

Additional file 1: Figure S1. The Ct-OATP1B3 mRNA expression T/N ratio in each pair of the colon and lung tissues. The T/N ratio of Ct-OATP1B3 mRNA was calculated in individual colon cancer (A) and lung cancer (B) patients who showed its positive expression, where the Ct-OATP1B3 mRNA expression value of the normal tissue was set to the baseline. Ct-OATP1B3 mRNA levels in normal tissues were tentatively corrected as 10^3^ copies/ng total RNA (identical to the QL value) if the mRNA level was the QL. The values obtained from each matched pair were connected by a line. Gray lines indicate the T/N ratios that were calculated using the corrected values, while the blue lines indicate the T/N ratios that were calculated using the original values. Click here for file

Additional file 2: Figure S2. Comparison between the Ct- and Lt-OATP1B3 mRNA levels in each colon and lung cancer tissues. Fold differences between Ct- and Lt-OATP1B3 mRNA levels were calculated in individual colon cancer (A) and lung cancer (B) patients who showed positive Ct-OATP1B3 mRNA expression in cancer tissue, where the Lt-OATP1B3 mRNA level was set to the baseline. The Lt-OATP1B3 mRNA levels in normal tissues were tentatively corrected as 10^3^ copies/ng total RNA (identical to the QL value) if the mRNA level was the QL. The values obtained from an individual cancer tissue were connected by a line. The gray lines indicate the fold differences that were calculated using the corrected values, while the blue lines indicate the fold differences that were calculated using the original values. Click here for file

Additional file 3: Figure S3. Examination of Ct-OATP1B3 protein expression in the soluble membrane fractions of colon cancer cells. Western blotting was performed using the soluble membrane fraction of LS180 or HCT116 cells with rabbit anti-OATP1B3 serum that was developed by immunizing a rabbit with the synthesized epitope peptide (LEFLNNGEHFVPSAGTD). A control rabbit serum was also used for comparison. The soluble membrane fraction of human liver tissue (HL) was used as a positive control. Na^+^/K^+^ ATPase was used as a membrane marker. Click here for file

Additional file 4: Figure S4. Examination of Ct-OATP1B3 protein expression in the whole cell lysates of colon cancer cells. Western blotting was performed using the whole cell lysates of LS180 or HCT116 cells with (A) rabbit polyclonal anti-OATP1B3 antibodies (Sigma) or (B) rabbit anti-OATP1B3 serum (developed in this study). The soluble membrane fraction of human liver tissue (HL) was used as a positive control. β-actin was used as a loading control. Click here for file

Additional file 5: Figure S5. Transient expression and functional analysis of Ct-OATP1B3 in HCT116 cells. (A) Immunohistochemistry was performed to examine the protein expression of each OATP1B3 isoform using HCT116 cells transiently transfected with the Lt1B3/p3.1 (Lt-1B3/HCT116), Ct1B3-C/pBapo (Ct-1B3-C/HCT116), Ct1B3-v1/pBapo (Ct-1B3-v1/HCT116), or an empty vector (mock/HCT116). The representative results that were obtained from there independent experiments are shown. (B) The CCK-8 (1 μM) uptake by Lt-1B3/HCT116, Ct-1B3-C/HCT116, Ct-1B3-v1/HCT116, or mock/HCT116 was examined by transport analysis in the absence (black bar) or presence (white bar) of BSP (100 μM). The uptake level of CCK-8 in Lt-1B3/HCT116, Ct-1B3-C/HCT116 or Ct-1B3-v1/HCT116 cells was represented as the relative ratio to that observed in mock/HCT116 cells. The experiment was performed three times in duplicate. Click here for file

Additional file 6: Figure S6. Stable expression and functional analysis of Ct-OATP1B3 in HEK293 cells. (A) Either Ct-OATP1B3 or Lt-OATP1B3 mRNA expression level in human pooled hepatocytes (Hep50), Lt-1B3/HEK, Ct-1B3-C/HEK, Ct-1B3-v1/HEK and mock/HEK, was determined by qPCR using the primer set that could detect all mRNA isoforms, and the results were normalized using those of GAPDH. Each mRNA expression level is shown as mean ± S.D. of percentages relative to the level of pooled human hepatocytes (100%). Experiments were performed three times in duplicate. N.D. indicates that the value was too low to be calculated. (B) The protein expression of either Ct-OATP1B3 or Lt-OATP1B3 in Ct-1B3-C/HEK, Ct-1B3-v1/HEK, Lt-1B3/HEK, and mock/HEK, was examined by Western blotting using the anti-OATP1B3 antibodies. The soluble membrane fraction of human liver tissue (HL) was used as a positive control. Na^+^/K^+^ ATPase was used for a plasma membrane marker. (C) The CCK-8 (1 μM, left) or E_2_G (0.5 μM, right) uptake by Lt-1B3/HEK, Ct-1B3-C/HEK, Ct-1B3-v1/HEK and mock/HEK was examined by transport analysis in the absence (black bar) or presence (white bar) of BSP (100 μM). The uptake level of each substrate in Lt-1B3/HEK, Ct-1B3-C/HEK or Ct-1B3-v1/HEK was represented as the relative ratio to that observed in mock/HEK cells. The experiment was performed three times in duplicate. Click here for file

Additional file 7: Figure S7. Receiver operating characteristic (ROC) analysis for Ct-OATP1B3 mRNA in colon cancer patients. To assess the diagnostic potency of Ct-OATP1B3 mRNA in terms of its ability to discriminate cancer tissues from normal tissues, the ROC curve was generated on the basis of Ct-OATP1B3 mRNA levels in the matched-pairs of colon cancer and normal tissues (n = 39). During this analysis, the mRNA levels under the QL value were set to 10^3^ copies/ng total RNA (identical to the QL value) and used in the calculation. The area under the ROC curve (AUC) along with its 95% confidence intervals (CI) was analyzed using Prism 6 (GraphPad Software, La Jolla, CA). The AUC is 0.930 (95% CI = 0.865-0.994; *P* <0.0001). Click here for file

Additional file 8: Supplemental materials and methods. Click here for file
