# Supplementary figures and images for: Unique expression features of cancer-type organic anion transporting polypeptide 1B3 mRNA expression in human colon and lung cancers
Source: Clin Transl Med. 2014 Nov 18;3:37. doi: 10.1186/s40169-014-0037-y (PMC4298695; doi:10.1186/s40169-014-0037-y)

**A**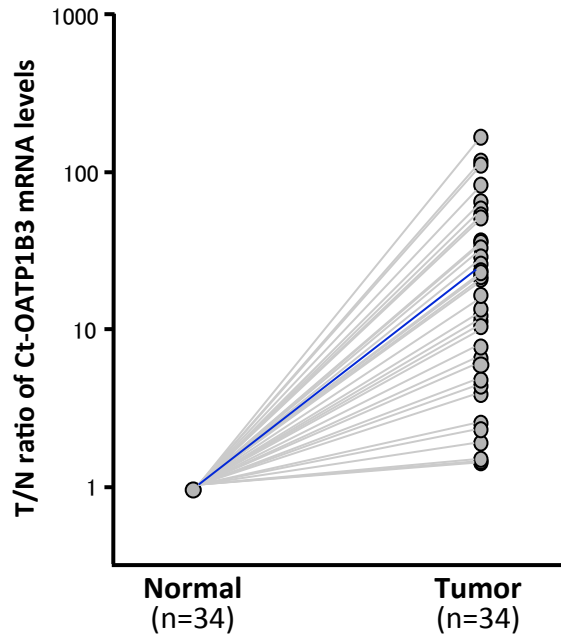**B**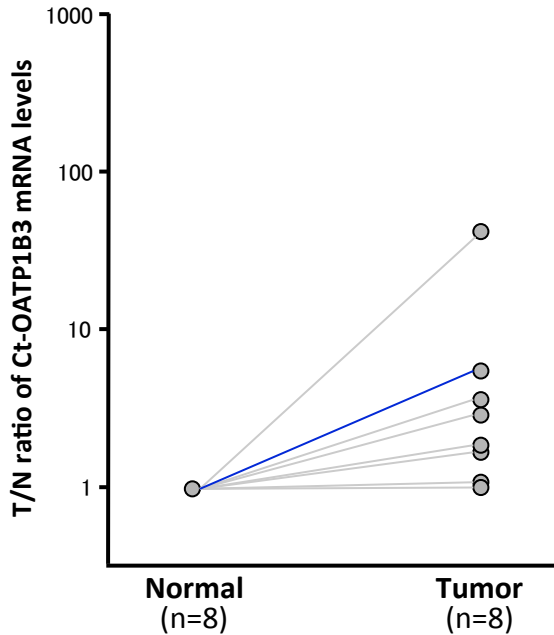

Figure S1

Supplement: Additional file 1: Figure S1. — The Ct-OATP1B3 mRNA expression T/N ratio in each pair of the colon and lung tissues. The T/N ratio of Ct-OATP1B3 mRNA was calculated in individual colon cancer (A) and lung cancer (B) patients who showed its positive expression, where the Ct-OATP1B3 mRNA expression value of the normal tissue was set to the baseline. Ct-OATP1B3 mRNA levels in normal tissues were tentatively corrected as 103 copies/ng total RNA (identical to the QL value) if the mRNA level was the QL. The values obtained from each matched pair were connected by a line. Gray lines indicate the T/N ratios that were calculated using the corrected values, while the blue lines indicate the T/N ratios that were calculated using the original values. [file s40169-014-0037-y-S1.pdf]

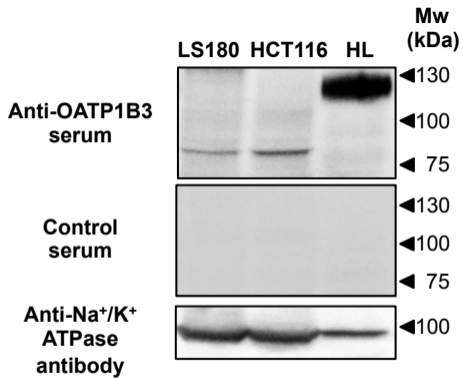

Figure S3

Supplement: Additional file 3: Figure S3. — Examination of Ct-OATP1B3 protein expression in the soluble membrane fractions of colon cancer cells. Western blotting was performed using the soluble membrane fraction of LS180 or HCT116 cells with rabbit anti-OATP1B3 serum that was developed by immunizing a rabbit with the synthesized epitope peptide (LEFLNNGEHFVPSAGTD). A control rabbit serum was also used for comparison. The soluble membrane fraction of human liver tissue (HL) was used as a positive control. Na+/K+ ATPase was used as a membrane marker. [file s40169-014-0037-y-S3.pdf]

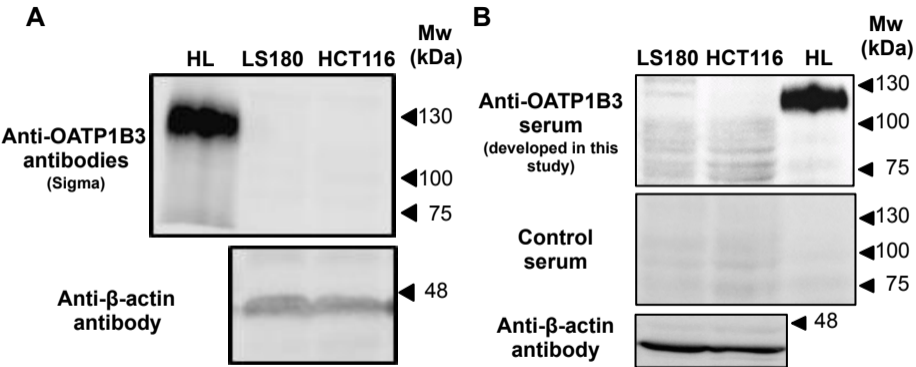

Figure S4

Supplement: Additional file 4: Figure S4. — Examination of Ct-OATP1B3 protein expression in the whole cell lysates of colon cancer cells. Western blotting was performed using the whole cell lysates of LS180 or HCT116 cells with (A) rabbit polyclonal anti-OATP1B3 antibodies (Sigma) or (B) rabbit anti-OATP1B3 serum (developed in this study). The soluble membrane fraction of human liver tissue (HL) was used as a positive control. β-actin was used as a loading control. [file s40169-014-0037-y-S4.pdf]

**A**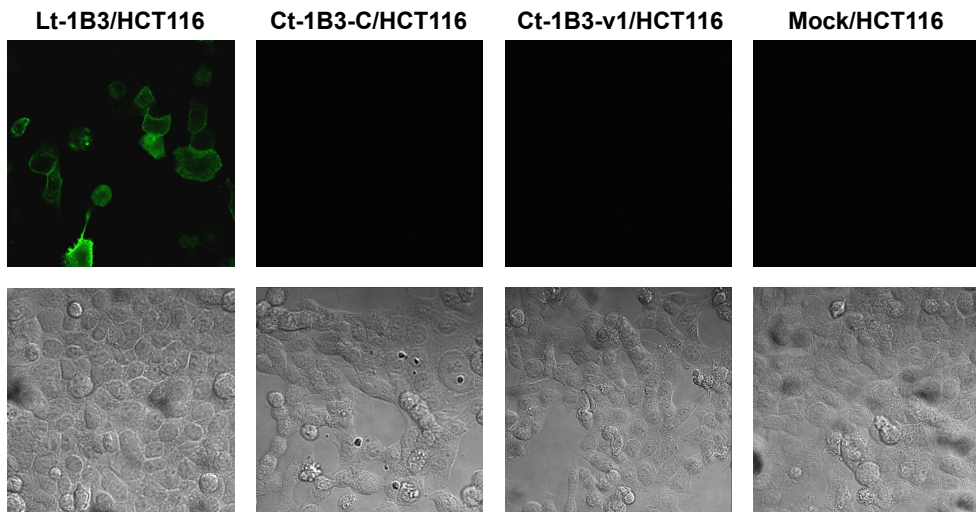**B**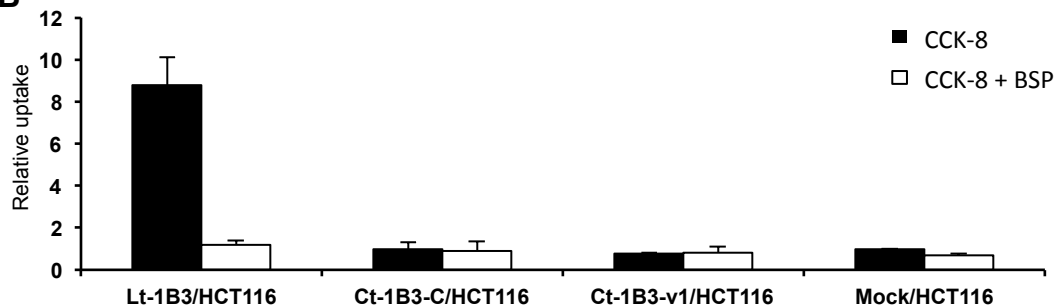

Figure S5

Supplement: Additional file 5: Figure S5. — Transient expression and functional analysis of Ct-OATP1B3 in HCT116 cells. (A) Immunohistochemistry was performed to examine the protein expression of each OATP1B3 isoform using HCT116 cells transiently transfected with the Lt1B3/p3.1 (Lt-1B3/HCT116), Ct1B3-C/pBapo (Ct-1B3-C/HCT116), Ct1B3-v1/pBapo (Ct-1B3-v1/HCT116), or an empty vector (mock/HCT116). The representative results that were obtained from there independent experiments are shown. (B) The CCK-8 (1 μM) uptake by Lt-1B3/HCT116, Ct-1B3-C/HCT116, Ct-1B3-v1/HCT116, or mock/HCT116 was examined by transport analysis in the absence (black bar) or presence (white bar) of BSP (100 μM). The uptake level of CCK-8 in Lt-1B3/HCT116, Ct-1B3-C/HCT116 or Ct-1B3-v1/HCT116 cells was represented as the relative ratio to that observed in mock/HCT116 cells. The experiment was performed three times in duplicate. [file s40169-014-0037-y-S5.pdf]

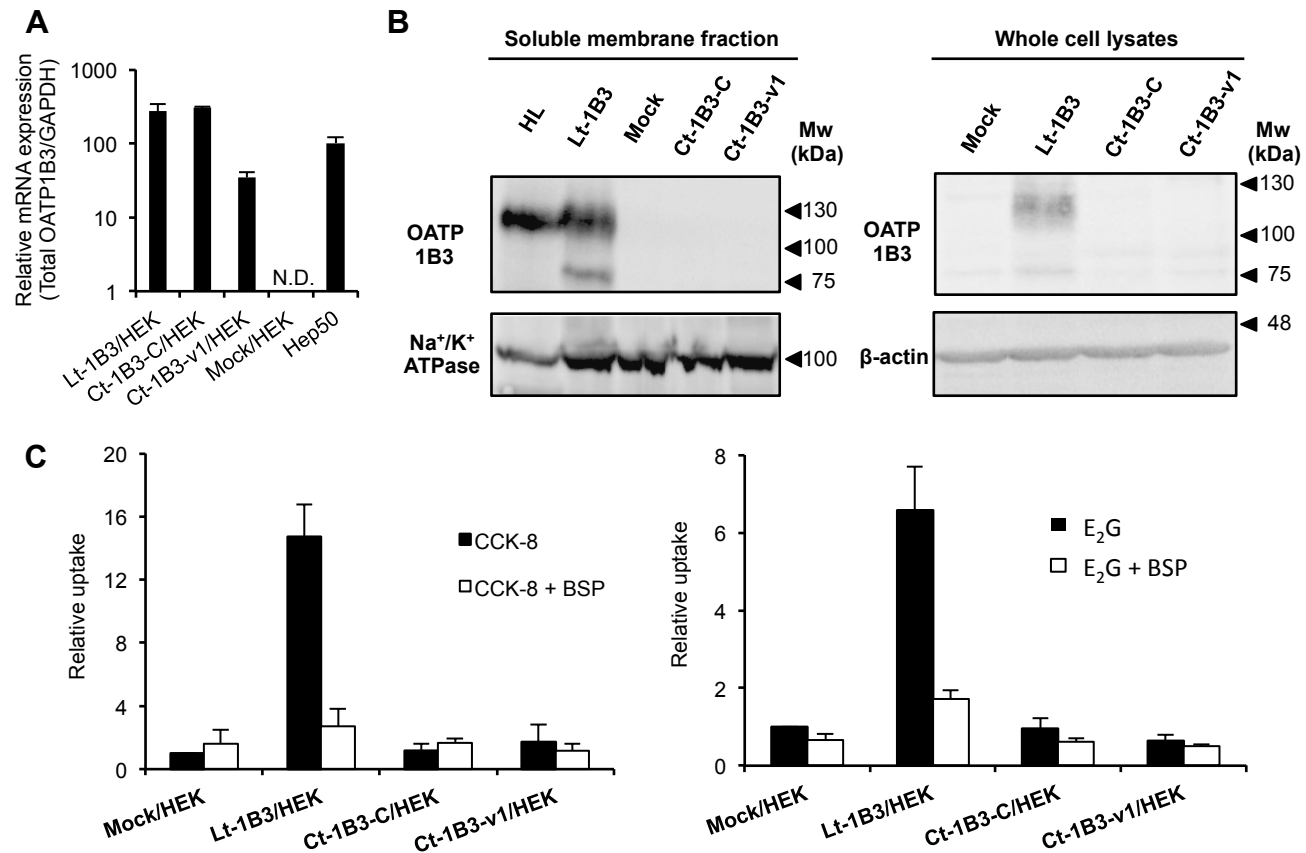

Figure S6

Supplement: Additional file 6: Figure S6. — Stable expression and functional analysis of Ct-OATP1B3 in HEK293 cells. (A) Either Ct-OATP1B3 or Lt-OATP1B3 mRNA expression level in human pooled hepatocytes (Hep50), Lt-1B3/HEK, Ct-1B3-C/HEK, Ct-1B3-v1/HEK and mock/HEK, was determined by qPCR using the primer set that could detect all mRNA isoforms, and the results were normalized using those of GAPDH. Each mRNA expression level is shown as mean ± S.D. of percentages relative to the level of pooled human hepatocytes (100%). Experiments were performed three times in duplicate. N.D. indicates that the value was too low to be calculated. (B) The protein expression of either Ct-OATP1B3 or Lt-OATP1B3 in Ct-1B3-C/HEK, Ct-1B3-v1/HEK, Lt-1B3/HEK, and mock/HEK, was examined by Western blotting using the anti-OATP1B3 antibodies. The soluble membrane fraction of human liver tissue (HL) was used as a positive control. Na+/K+ ATPase was used for a plasma membrane marker. (C) The CCK-8 (1 μM, left) or E2G (0.5 μM, right) uptake by Lt-1B3/HEK, Ct-1B3-C/HEK, Ct-1B3-v1/HEK and mock/HEK was examined by transport analysis in the absence (black bar) or presence (white bar) of BSP (100 μM). The uptake level of each substrate in Lt-1B3/HEK, Ct-1B3-C/HEK or Ct-1B3-v1/HEK was represented as the relative ratio to that observed in mock/HEK cells. The experiment was performed three times in duplicate. [file s40169-014-0037-y-S6.pdf]

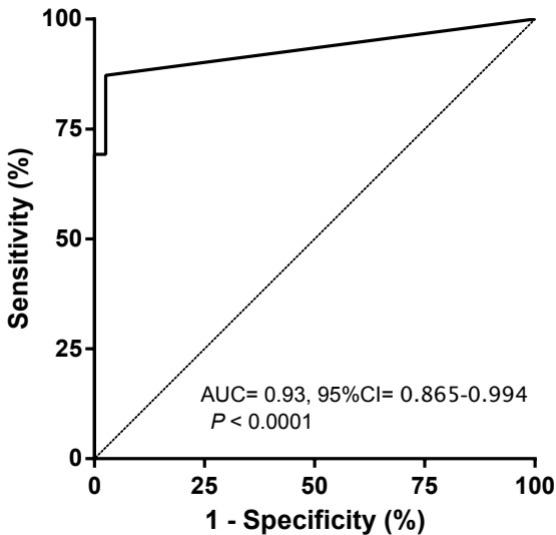

Figure S7

Supplement: Additional file 7: Figure S7. — Receiver operating characteristic (ROC) analysis for Ct-OATP1B3 mRNA in colon cancer patients. To assess the diagnostic potency of Ct-OATP1B3 mRNA in terms of its ability to discriminate cancer tissues from normal tissues, the ROC curve was generated on the basis of Ct-OATP1B3 mRNA levels in the matched-pairs of colon cancer and normal tissues (n = 39). During this analysis, the mRNA levels under the QL value were set to 103 copies/ng total RNA (identical to the QL value) and used in the calculation. The area under the ROC curve (AUC) along with its 95% confidence intervals (CI) was analyzed using Prism 6 (GraphPad Software, La Jolla, CA). The AUC is 0.930 (95% CI = 0.865-0.994; P <0.0001). [file s40169-014-0037-y-S7.pdf]
