# Supplementary material for: Unique expression features of cancer-type organic anion transporting polypeptide 1B3 mRNA expression in human colon and lung cancers
Source: Clin Transl Med. 2014 Nov 18;3:37. doi: 10.1186/s40169-014-0037-y (PMC4298695; doi:10.1186/s40169-014-0037-y)
Supplement: Additional file 2: Figure S2. — Comparison between the Ct- and Lt-OATP1B3 mRNA levels in each colon and lung cancer tissues. Fold differences between Ct- and Lt-OATP1B3 mRNA levels were calculated in individual colon cancer (A) and lung cancer (B) patients who showed positive Ct-OATP1B3 mRNA expression in cancer tissue, where the Lt-OATP1B3 mRNA level was set to the baseline. The Lt-OATP1B3 mRNA levels in normal tissues were tentatively corrected as 103 copies/ng total RNA (identical to the QL value) if the mRNA level was the QL. The values obtained from an individual cancer tissue were connected by a line. The gray lines indicate the fold differences that were calculated using the corrected values, while the blue lines indicate the fold differences that were calculated using the original values. [file s40169-014-0037-y-S2.pdf]

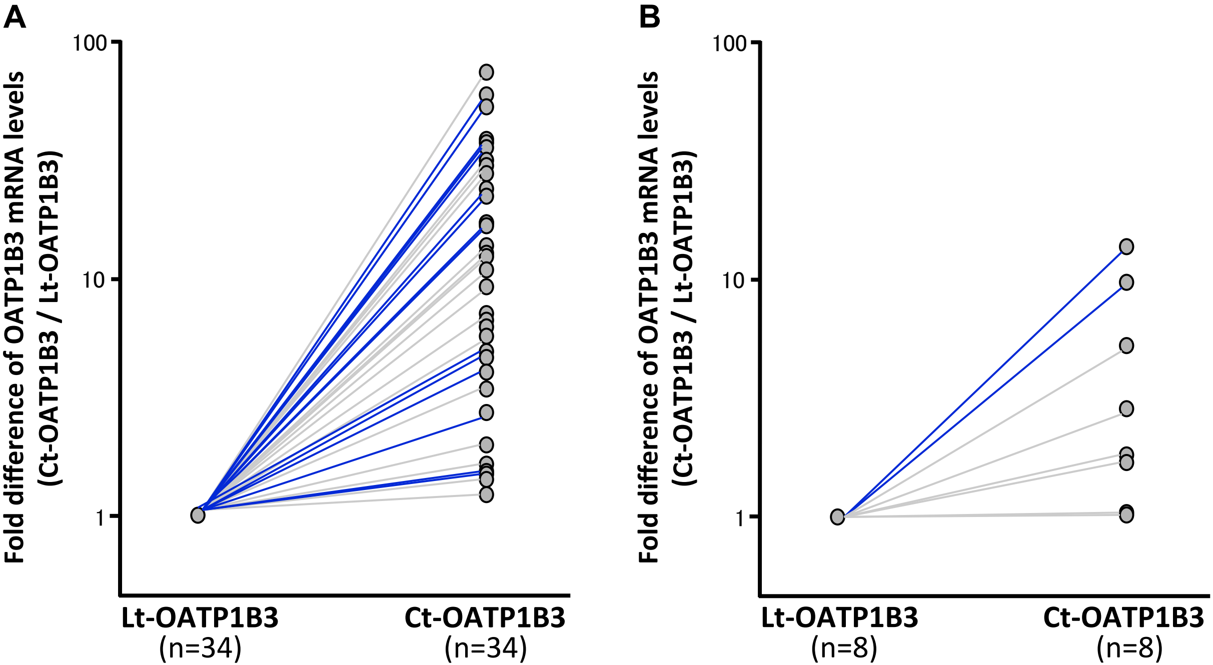
A
B
100
10
1
Fold difference of OATP1B3 mRNA levels
(Ct-OATP1B3 / Lt-OATP1B3)
Lt-OATP1B3
(n=34)
Ct-OATP1B3
(n=34)
Lt-OATP1B3
(n=8)
Ct-OATP1B3
(n=8)

Figure S2
