# Supplementary material for: Unique expression features of cancer-type organic anion transporting polypeptide 1B3 mRNA expression in human colon and lung cancers
Source: Clin Transl Med. 2014 Nov 18;3:37. doi: 10.1186/s40169-014-0037-y (PMC4298695; doi:10.1186/s40169-014-0037-y)
Supplement: Additional file 8: — Supplemental materials and methods. [file s40169-014-0037-y-S8.docx]

**Additional file 8: Supplemental materials and methods**

**Rabbit anti-OATP1B3 serum preparation**

Rabbit anti-OATP1B3 serum was collected from a rabbit immunized with the synthesized Lt-OATP1B3 C-terminus peptide, 671-687 LEFLNNGEHFVPSAGTD (Sigma). Additionally, a control serum was collected from the rabbit prior to immunization.

**Cells and cell culture**

The human kidney embryonic 293 (HEK293) cells were obtained from Human Health Science Research Bank (Osaka, Japan).

HEK293 cells were maintained in Dulbecco's Modified Eagle's Medium (Wako, Osaka, Japan) supplemented with 10% (v/v) heat-inactivated fetal bovine serum and antibiotics. The cells were grown at 37ºC with 5% CO_2_.

**Preparation of OATP1B3 expression plasmids**

Ct-OATP1B3 cDNA containing the entire sequence from transcription start site to stop codon was subcloned into the pcDNA3.1(-)Neo (Ct1B3/p3.1) (Life Technologies, Carlsbad, CA) from the Ct-OATP1B3 expression plasmid (Ct1B3/pTOPO), which had been prepared in our previous study [5]. Ct-OATP1B3-C (lacking the N-terminal 47 amino acids of Lt-OATP1B3, Fig. 1) cDNA was amplified by polymerase chain reaction (PCR) using the Ct1B3/p3.1 as a template with the primer set (5’-GTGGATCCATTATGAAAATTTCCATCACT-3’ and 5’-GCTCTAGAGCAATGTTAGTTGGCAGCAGCA-3’) and subcloned into the pBapo-CMVpur (Ct1B3-C/pBapo) (Takara Bio, Siga, Japan). Similarly, Ct-OATP1B3-v1 (lacking the N-terminal 28 amino acids of Lt-OATP1B3, Fig. 1) cDNA was cloned into the pBapo-CMVpur (Ct1B3-v1/pBapo) using the primer set (5’-GTGGATCCTAGCAGATGTTCTTGGCAGCCC-3’ and 5’- GCTCTAGAGCAATGTTAGTTGGCAGCAGCA-3’). The preparation of Lt-OATP1B3 expression plasmid (Lt1B3/p3.1) was described in the previous report [18].

**Development of HEK293 cells stably expressing each OATP1B3 isoform**

The Ct1B3-C/pBapo or Ct1B3-v1/pBapo was transfected into HEK293 cells using Lipofectamine LTX (Life Technologies) according to the manufacturer’s protocol. The cells transfected with Ct1B3-C/pBapo (Ct-1B3-C/HEK) or Ct1B3-v1/pBapo (Ct-1B3-v1/HEK) were selected by supplying 0.6 μg/mL puromycin into the culture medium. Using the same procedure, HEK293 carrying empty pcDNA3.1(-)Neo or empty pBapo-CMVpur were developed (mock/HEK). Single cell cloning of Ct-1B3-C/HEK was performed by a colony formation method. HEK293 cells stably expressing Lt-OATP1B3 (Lt-1B3/HEK) were developed in our previous study [18].

Transport assay was performed using the method described in the method section of the main text. Briefly, one day after the cells were seeded, they were exposed to sodium butyrate (10 mM, Sigma) for 24 hours, after which the transport assay was performed using CCK-8 and E_2_G as substrates.

**Immunocytochemistry**

Immunocytochemistry was performed essentially using the methods described in our previous report [18]. Briefly, HCT116 cells were seeded on the collagen-coated coverslip, after which the cells were transfected with the Lt1B3/p3.1, Ct1B3-C/pBapo, Ct1B3-v1/pBapo, or an empty vector using MultiFectam transfection reagent according to the manufacturer’s protocol (Promega, Fitchburg, WI). Forty-eight hours after transfection, the cells were fixed and permeabilized with BD Cytofix / Cytoperm kit (BD Bioscience, Franklin Lakes, NJ). After blocking with 3% bovine serum albumin, the cells were incubated with rabbit polyclonal anti-OATP1B3 antibodies (200-fold dilution, Sigma). The secondary antibody used was Alexa Fluor 488-conjugated donkey anti-rabbit antibody (200-fold dilution, Life Technologies). Immunofluorescence was analyzed using confocal laser scanning immunofluorescence microscopy (LSM 710; ZEISS, Oberkochen, Germany).

**Transient expression and functional analysis of Ct-OATP1B3 in HCT116 cells**

HCT116 cells were transfected with the Lt1B3/p3.1, Ct1B3-C/pBapo, Ct1B3-v1/pBapo, or an empty vector using MultiFectam transfection reagent according to the manufacturer’s protocol. Transport assay was performed using the same method as that described above.
